# The burden of atrial fibrillation/flutter in the Middle East and North Africa region and its associated risk factors from 1990 to 2019

**DOI:** 10.1186/s12872-024-04019-2

**Published:** 2024-07-16

**Authors:** Mohammad Yaghoubi, Parvaneh Hamian Roumiani, Fateme Nozari, Saba Simiyari, Alireza Azarboo, Mahgol Sadat Hassan Zadeh Tabatabaei, Mohamad Mehdi Khadembashiri, Mohammad Amin Khadembashiri

**Affiliations:** 1 Cardiology Department, Azerbaijan Medical University, Baku, Azerbaijan; 2 Cardiology Department, Tehran University of Medical Sciences, Tehran, Iran; 3 Rajaie Cardiovascular Medical and Research Center, School of Medicine, Iran University of Medical Sciences, Tehran, Iran; 4 School of Medicine, Tehran University of Medical Sciences, Tehran, Iran; 5 Sina Trauma and Surgery Research Center, Tehran University of Medical Sciences, Tehran, Iran

**Keywords:** Atrial fibrillation/atrial flutter, Disease burden, Age-standardized rate, Risk factors

## Abstract

**Background:**

Atrial fibrillation and flutter (AFF) are the most common cardiac arrhythmias globally, contributing to substantial morbidity and mortality. The Middle East and North Africa (MENA) region face unique challenges in managing cardiovascular diseases, including AFF, due to diverse sociodemographic factors and healthcare infrastructure variability. This study aims to comprehensively evaluate the burden of AFF in MENA from 1990 to 2019.

**Methods:**

Data were obtained from the Global Burden of Diseases Study 2019, a comprehensive source incorporating diverse data inputs. The study collected global, regional, and national Age-Standardized Incidence Rate (ASIR), Age-Standardized Mortality Rate (ASMR), and Age-Standardized Disability-Adjusted Rate (ASDR), Mortality across sex, age groups, and years. LOESS regression was employed to determine the relationship between age-standardized rates attributed to AFF and Socio-Demographic Index (SDI).

**Results:**

The study found minimal change in ASIR of AFF in MENA from 1990 to 2019, with a slight increase observed in ASMR and ASDR during the same period. Notably, AFF burden was consistently higher in females compared to males, with age showing a direct positive relationship with AFF burden. Iraq, Iran, and Turkey exhibited the highest ASIR, while Qatar, Bahrain, and Oman had the highest ASMR and ASDR in 2019. Conversely, Kuwait, Libya, and Turkey displayed the lowest ASMR and ASDR rates.

**Conclusion:**

This study underscores the persistent burden of AFF in MENA and identifies significant disparities across countries. High systolic blood pressure emerged as a prominent risk factor for mortality in AFF patients. Findings provide crucial insights for policy-making efforts, resource allocation, and intervention strategies aimed at reducing the burden of cardiovascular diseases in the MENA region.

**Supplementary Information:**

The online version contains supplementary material available at 10.1186/s12872-024-04019-2.

## Introduction

Atrial fibrillation and atrial flutter (AF/AFL) represent prevalent and enduring forms of tachyarrhythmia, profoundly affecting individuals’ health and quality of life [1–3]. The prevalence and incidence of these conditions vary widely among countries, influenced by an array of factors such as age, sex, body mass index (BMI), lifestyle choices including smoking and alcohol consumption, systolic blood pressure, a diet high in sodium, lead exposure, genetic predisposition, socioeconomic status, and educational background [3–6]. AF/AFL exhibits a notable increase in prevalence and incidence with age, with men showing a higher prevalence than women, particularly in younger age groups. However, this trend reverses in individuals over 75 years old [7]. Notably, regions with lower socioeconomic development indices are witnessing a concerning rise in AF/AFL prevalence [8].

AF/AFL not only impairs quality of life but also escalates the risk of morbidity and mortality, often leading to severe complications like heart attack, heart failure, stroke, dementia, and cognitive decline [9, 10]. The coexistence of AF/AFL with other conditions exacerbates mortality risks [11], with both cardiac and non-cardiac deaths, including sudden cardiac death, being heightened [12].

Hospitalization rates due to AF/AFL are steadily increasing due to a complex interplay of genetic, biological, and environmental factors [13]. Despite advancements in treatment, the global burden of AF/AFL continues to rise significantly, with an estimated 59.7 million people affected worldwide in 2019, nearly double the estimated cases from 2010 [5, 13]. Projections suggest a troubling trend of escalating mortality and morbidity rates associated with AF/AFL, with an anticipated tripling of these rates over the next three decades [14]. By 2030 to 2034, over 16 million incidences of AF/AFL are expected annually [15]. This necessitates sustained efforts in surveillance, prevention, and treatment.

Given the preventable nature of many risk factors associated with AF/AFL, there is an urgent need for targeted interventions, particularly in regions experiencing a high or growing burden of the disease [8]. A precise assessment of the current burden and underlying risk factors specific to the Middle East and North Africa (MENA) region is crucial for devising effective strategies to combat AF/AFL and its complications. While previous studies from the Global Burden of Diseases, Injuries, and Risk Factors Study (GBD) 2019 have addressed the global impact of AF/AFL [7, 8, 15, 16], to our knowledge, there lacks an updated evaluation providing a comprehensive analysis of various factors encompassing prevalence, incidence rates, Disability-Adjusted Life Years (DALYs), mortality rates, and significant risk factors specific to AF/AFL in MENA over time. Thus, this study explores the burden and associated risk factors of AF/AFL in MENA between 1990 and 2019, leveraging data from GBD provided by the Institute of Health Metrics and Evaluation (IHME).

## Materials and methods

### Source

Using the Global Health Data Exchange query tool created by GBD partners, the Institute of Health Metrics and Evaluation (IHME) gathered available data, standardized disease criteria, and other relevant statistics. The GBD Study assesses the annual burden of 369 diseases, 87 risk factors, and injuries across 204 countries and territories from 1990 to 2019 [17, 18]. The data were obtained from the GBD Study, a comprehensive and standardized source that incorporates a range of data inputs including hospital records, surveys, censuses, vital registration, systematic reviews, and disease-specific registries. To find the percentage of each component that correlates to a specific outcome, clinical record data were collected [17, 18]. A variety of variables, such as age, sex, and geography, are used in the statistical models of the GBD Study. To handle incomplete or missing reports, the GBD Study makes use of advanced statistical models, including DisMod-MR 2.1, a reliable Bayesian meta-regression tool for improving estimation accuracy. Additionally, uniformity across different data sources is ensured by the implementation of standardized cleaning processes [17, 18].

### Data and indices

In this study, we collected global, regional, and national counts and rates of AF/AFL incidence, mortality, and DALYs across sex, age groups, and years from 1990 to 2019. The data collection encompassed MENA countries, including Afghanistan, Algeria, Bahrain, Egypt, Iran, Iraq, Jordan, Kuwait, Lebanon, Libya, Morocco, Oman, Palestine, Qatar, Saudi Arabia, Sudan, Syria, Tunisia, Turkey, United Arab Emirates, and Yemen. In the GBD Study, the total of years lived with disability (YLDs) and years of life lost (YLLs) is known as disability-adjusted life years (DALYs), which is a metric used to assess a population’s overall health status for a particular region, sex, year, and age combination [17, 18]. The Socio-demographic Index (SDI) is another tool used by the GBD Study to evaluate the state of national development. The SDI values, which are computed by combining three indicators—income per capita, the fertility rate of people under 25, and the mean years of education of those under 15—range from 0 to 1, with lower values denoting lower development [19].

### Statistical analysis

Estimated Age-Standardized Incidence Rate (ASIR), and Age-Standardized Mortality Rate (ASMR), Age-Standardized Disability-Adjusted Rate (ASDR) i.e., DALY were reported along with the 95% uncertainty intervals. Percent of change (%C) from 1990 to 2019 were also computed. We used Python 3.8 to construct all figures and choropleth maps. To determine the relationship between age-standardized rates attributed to AFF and SDI, we used locally estimated scatterplot smoothing (LOESS) regression to draw a predicted value line.

## Results

### Burden of atrial fibrillation and flutter in MENA

**Fig. 1:**
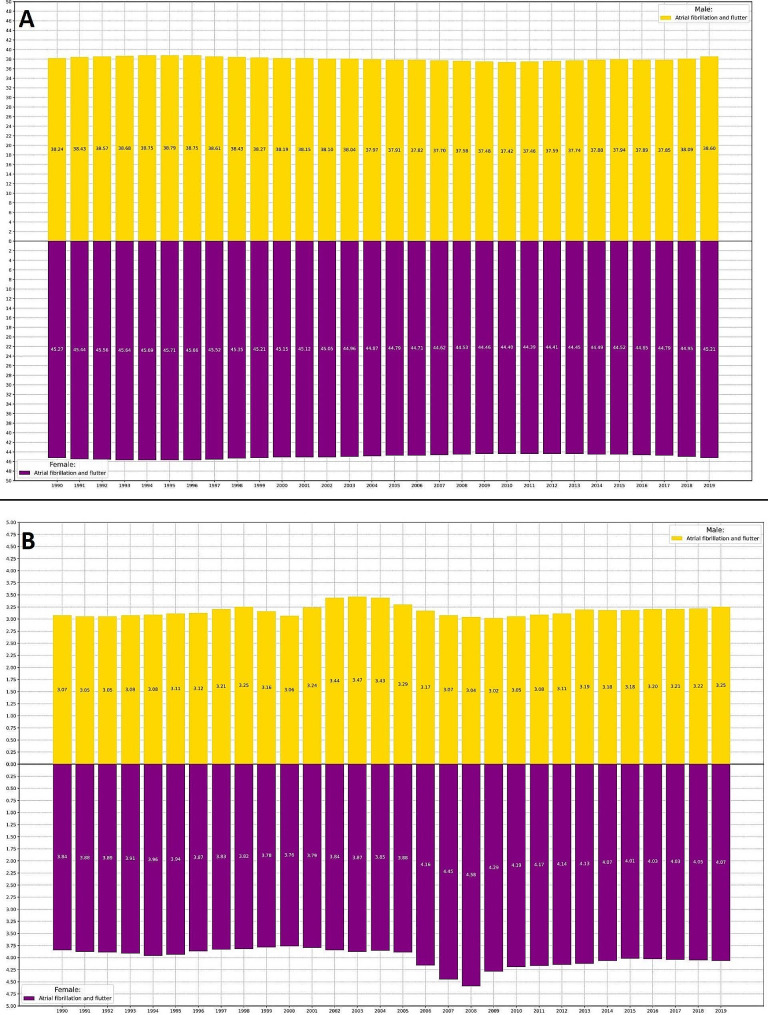
Age-standardized Incidence (**A**) and Mortality (**B**) Rate of AFF from 1990 to 2019

Regional ASIR owing to AFF changed very slightly in the Middle East and North Africa (MENA) from 41.81 [31.63 to 53.41] in 1990 to 41.93 [31.66 to 53.56] in 2019 (%C ASIR = 0.29%) (Fig. 1A). But from 1990 (3.48 [2.67 to 4.11]) to 2019 (3.66 [3.07 to 4.33]) (%C ASMR = 5.17%), there was a slight increase in ASMR (Fig. 1B). Furthermore, from 1990 (79.91 [63.99 to 99.66]) to 2019 (81.61 [65.10 to 100.78]) (%C ASDR = 2.13%), it was noted that ASDR had somewhat grown (Figure S1). A small increasing slope was observed in ASIR and ASDR between 2010 and 2019, despite the fact that the temporal trend stayed constant throughout the course of 30 years. The slope was noted for ASMR from 2014 to 2019.

### Atrial fibrillation and flutter across sex and age groups

**Fig. 2:**
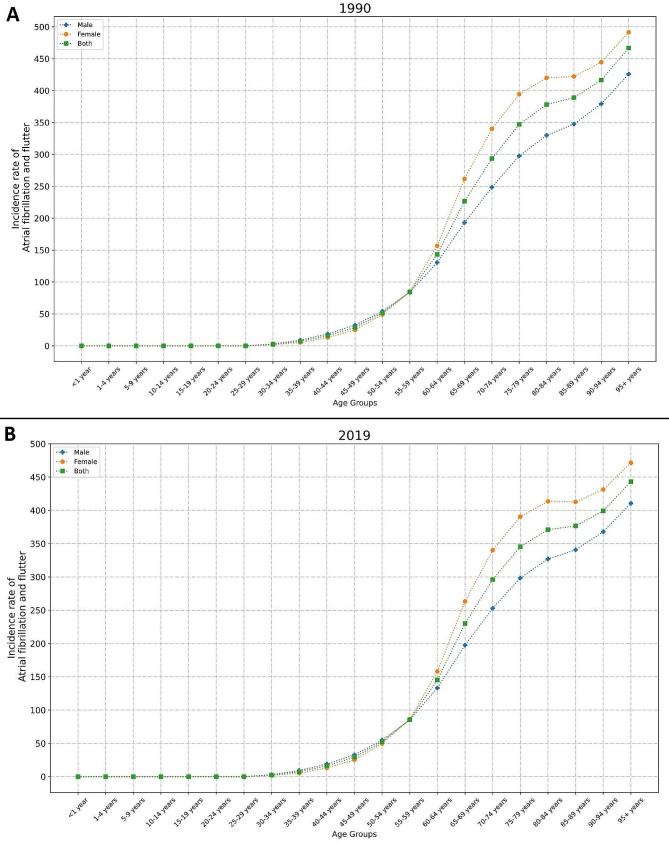
Incidence Rate of AFF from in different age groups in 1990 (**A**) and 2019 (**B**)

**Fig. 3:**
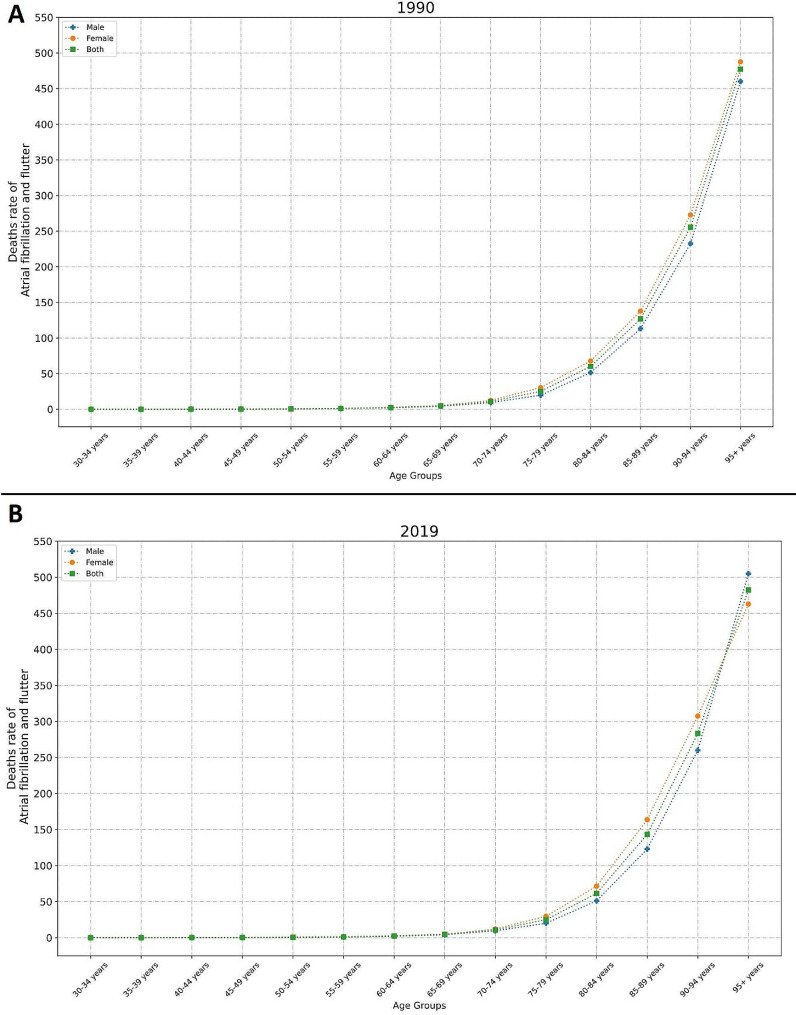
Mortality Rate of AFF from in different age groups in 1990 (**A**) and 2019 (**B**)

In general, ASIR, ASMR, and ASDR attributable to AFF were significantly higher in females compared to males either in 1990 (ASIR _Female_ = 45.27 [33.84 to 58.08], ASIR _Male_ = 38.24 [29.17 to 48.94], ASMR _Female_ = 3.84 [2.77 to 4.94], ASMR _Male_ = 3.07 [2.15 to 4.23], ASDR _Female_ = 86.04 [65.79 to 109.21], ASDR _Male_ = 73.12 [55.34 to 95.17]) or 2019 (ASIR _Female_ = 45.21 [33.83 to 58.03], ASIR _Male_ = 38.60 [29.41 to 49.55], ASMR _Female_ = 4.07 [3.39 to 4.83], ASMR _Male_ = 3.25 [2.54 to 4.00], ASDR _Female_ = 88.01 [70.33 to 109.65], ASDR _Male_ = 75.10 [58.52 to 93.83]). A steady trend in ASIR and a slight increasing slope in ASMR and ASDR was observed in both males and females across these thirty years. The highest incidence rate was observed in males + 95 years old, in both 1990 (Fig. 2A) and 2019 (Fig. 2B). However, in terms of mortality and DALYs, females + 95 years old surpassed males from 1990 (Fig. 3A, S2A) to 2019 (Fig. 3B, S2B). Age seemed to have a direct positive relationship with AFF’s burden.

### National burden of atrial fibrillation and flutter

Iraq (43.84 [33.03 to 56.02]), Iran (42.76 [32.42 to 55.06]), and Turkey (42.63 [32.10 to 54.55]) had the highest ASIR due to AFF in 2019. On the other hand, Yemen (39.69 [30.03 to 51.24]), Saudi Arabia (39.79 [30.10 to 50.76]), and Palestine (40.09 [30.41 to 51.31]) had the lowest. The highest increase in ASIR was observed in Oman (%C = 7.0%), Afghanistan (%C = 3.6%), and Sudan (%C = 3.5%). Nonetheless, ASIR was considerably reduced in United Arab Emirates (%C = -5.1%), Qatar (%C = -3.4%), and Bahrain (%C = -2.9%) from 1990 (Table 1).

**Table 1:** Age-Standardized Incidence rate (ASIR) for atrial fibrillation and flutter in Middle East and North Africa

Location Name	1990 Number (All ages)	2019 Number (All ages)	1990 ASIR [95%CI]	2019 ASIR [95%CI]	C% (Percentage Change)
Jordan	506.21 [384.8 to 652.76]	2566.91 [1962.57 to 3276.86]	41.16 [30.9 to 53.02]	40.83 [30.84 to 52.28]	407.08%
Bahrain	74.26 [57.18 to 94.79]	433.34 [324.9 to 565.32]	43.54 [32.94 to 56.27]	42.26 [31.85 to 54.76]	483.52%
Iran (Islamic Republic of)	10318.24 [7742.96 to 13431.73]	30246.55 [23219.46 to 38550.89]	42.83 [32.58 to 54.95]	42.76 [32.42 to 55.06]	193.14%
North Africa and Middle East	67246.31 [51251.76 to 86435.36]	173920.92 [133004.26 to 221691.64]	41.81 [31.63 to 53.41]	41.93 [31.66 to 53.56]	158.63%
Syrian Arab Republic	2025.32 [1550.78 to 2606.96]	4919.33 [3676.08 to 6345.19]	41.16 [31.04 to 53.38]	41.11 [30.95 to 52.75]	142.89%
Libya	747.88 [575.49 to 972.08]	2066.15 [1589.16 to 2605.15]	42.29 [31.85 to 54.71]	42.37 [31.72 to 54.56]	176.27%
Kuwait	243.5 [190.69 to 307.65]	1077.52 [838.35 to 1352.3]	41.36 [31.35 to 52.93]	41.51 [31.31 to 53.18]	342.51%
Oman	246.24 [188.9 to 316.56]	745.42 [575.75 to 943.92]	39.82 [30.08 to 51.53]	42.61 [32.31 to 54.88]	202.72%
United Arab Emirates	195.85 [148.63 to 248.26]	1842.87 [1368.31 to 2395.34]	44.0 [33.32 to 56.23]	41.75 [31.45 to 53.07]	840.96%
Tunisia	1923.53 [1454.4 to 2500.3]	5030.8 [3799.78 to 6503.53]	39.94 [30.01 to 51.41]	40.51 [30.45 to 52.06]	161.54%
Algeria	4714.45 [3523.23 to 6130.18]	13206.53 [10041.51 to 17165.11]	40.13 [30.36 to 51.57]	40.5 [30.55 to 52.6]	180.13%
Morocco	5568.34 [4200.21 to 7222.17]	12756.76 [9679.9 to 16336.57]	43.09 [32.32 to 55.44]	42.5 [31.99 to 54.68]	129.09%
Egypt	11126.05 [8468.74 to 14377.18]	24804.67 [18787.64 to 31731.07]	40.87 [30.89 to 52.32]	41.12 [30.92 to 52.81]	122.94%
Qatar	50.49 [38.35 to 64.07]	454.86 [341.22 to 587.72]	42.26 [31.97 to 54.24]	40.83 [31.1 to 52.17]	800.91%
Sudan	3552.43 [2678.95 to 4550.57]	7375.65 [5631.55 to 9398.21]	40.39 [30.36 to 51.77]	41.83 [31.36 to 53.78]	107.62%
Iraq	3289.47 [2501.31 to 4235.11]	9517.84 [7285.67 to 12097.23]	44.49 [33.18 to 57.43]	43.84 [33.03 to 56.02]	189.34%
Global	2313539.87 [1764441.36 to 2950591.83]	4720323.57 [3644331.37 to 5961597.14]	58.54 [44.92 to 74.24]	57.09 [44.07 to 71.9]	104.03%
Lebanon	911.68 [687.34 to 1181.57]	2210.11 [1652.29 to 2847.01]	41.93 [31.62 to 54.11]	42.56 [31.75 to 54.97]	142.42%
Afghanistan	2645.64 [1984.84 to 3437.86]	4585.54 [3474.26 to 5854.15]	39.42 [29.98 to 50.72]	40.85 [30.96 to 52.69]	73.32%
Saudi Arabia	2180.27 [1692.15 to 2766.04]	6855.75 [5263.92 to 8686.57]	39.45 [29.83 to 50.49]	39.79 [30.1 to 50.76]	214.45%
Palestine	329.02 [250.89 to 424.0]	893.5 [685.24 to 1144.31]	39.86 [29.96 to 51.23]	40.09 [30.41 to 51.31]	171.57%
Yemen	1784.82 [1353.81 to 2294.88]	4945.83 [3813.3 to 6327.68]	40.0 [30.22 to 51.22]	39.69 [30.03 to 51.24]	177.1%
Türkiye	14767.4 [11249.84 to 18980.11]	37208.3 [27994.35 to 47644.58]	43.33 [32.8 to 55.63]	42.63 [32.1 to 54.55]	151.96%

The three nations with the highest ASMR due to AFF in 2019 were Qatar (12.11 [9.13 to 16.76]), Bahrain (10.65 [8.28 to 12.78]), and Oman (8.38 [5.14 to 10.31]). With the lowest ASMRs, however, were Kuwait (2.21 [1.67 to 3.04]), Libya (2.66 [1.78 to 3.63]), and Turkey (3.15 [2.43 to 3.97]). Bahrain (%C = 131.0%), Morocco (%C = 42.3%), and Iraq (%C = 28.9%) saw the largest increases in ASMR. However, when compared to 1990, ASMR was significantly lower in Turkey (%C = -16.8%), United Arab Emirates (%C = -2.7%), and Kuwait (%C = -2.0%) (Table 2).

**Table 2:** Age-Standardized Mortality Rate (ASMR) for atrial fibrillation and flutter in Middle East and North Africa

Location Name	1990 Number (All ages)	2019 Number (All ages)	1990 ASMR [95%CI]	2019 ASMR [95%CI]	C% (Percentage Change)
Global	117037.99 [103695.27 to 138452.31]	315336.77 [267964.21 to 361013.8]	4.29 [3.73 to 5.09]	4.38 [3.7 to 5.05]	169.43%
Lebanon	58.24 [45.66 to 72.83]	196.42 [124.04 to 255.14]	4.05 [3.15 to 5.12]	4.07 [2.57 to 5.27]	237.24%
Libya	32.67 [23.12 to 45.24]	103.91 [68.97 to 142.43]	2.28 [1.59 to 3.14]	2.66 [1.78 to 3.63]	218.08%
Algeria	247.73 [196.28 to 322.76]	966.89 [739.66 to 1230.25]	4.79 [3.69 to 6.43]	4.87 [3.67 to 6.11]	290.31%
Morocco	248.51 [174.02 to 308.49]	874.38 [664.61 to 1082.18]	3.07 [2.06 to 3.89]	4.37 [3.2 to 5.55]	251.85%
Palestine	24.64 [17.7 to 32.07]	63.87 [52.25 to 89.15]	3.88 [2.78 to 5.07]	4.64 [3.74 to 6.6]	159.17%
Saudi Arabia	129.69 [80.64 to 175.05]	300.84 [235.69 to 384.02]	3.96 [2.39 to 5.41]	4.29 [3.35 to 5.29]	131.96%
Qatar	4.67 [3.27 to 5.85]	21.19 [15.46 to 28.28]	12.02 [8.41 to 15.29]	12.12 [9.13 to 16.76]	353.77%
North Africa and Middle East	3462.66 [2708.46 to 3995.89]	10504.69 [8921.76 to 12764.88]	3.48 [2.67 to 4.11]	3.66 [3.07 to 4.33]	203.37%
Oman	17.19 [10.67 to 22.71]	41.08 [32.0 to 49.44]	7.45 [4.23 to 9.92]	8.38 [5.14 to 10.31]	138.92%
Egypt	529.84 [400.09 to 694.34]	1311.05 [852.93 to 1799.86]	3.36 [2.49 to 4.38]	3.93 [2.51 to 5.38]	147.44%
Bahrain	3.53 [3.01 to 4.68]	35.29 [25.84 to 43.79]	4.61 [3.88 to 6.68]	10.65 [8.28 to 12.78]	900.72%
Tunisia	93.26 [74.7 to 114.4]	383.37 [279.69 to 502.41]	3.38 [2.68 to 4.15]	3.9 [2.83 to 5.07]	311.07%
Türkiye	979.23 [740.28 to 1202.62]	2416.1 [1885.67 to 3072.99]	3.78 [2.79 to 4.72]	3.15 [2.43 to 3.97]	146.74%
Syrian Arab Republic	139.72 [106.19 to 172.37]	296.78 [218.34 to 385.21]	3.79 [2.85 to 4.67]	4.63 [3.35 to 5.95]	112.41%
Iraq	197.63 [145.67 to 308.2]	630.49 [480.69 to 1022.37]	3.44 [2.49 to 5.35]	4.43 [3.37 to 7.52]	219.03%
Jordan	30.29 [23.69 to 36.72]	138.66 [112.79 to 163.43]	4.47 [3.37 to 5.49]	4.39 [3.44 to 5.19]	357.76%
Yemen	73.82 [47.21 to 104.35]	254.81 [200.89 to 333.85]	2.91 [1.81 to 4.11]	3.34 [2.63 to 4.32]	245.17%
United Arab Emirates	5.39 [3.3 to 8.64]	38.22 [20.53 to 71.81]	4.23 [2.51 to 6.55]	4.12 [2.25 to 7.16]	609.36%
Afghanistan	113.96 [74.77 to 159.2]	216.48 [158.95 to 305.66]	2.69 [1.69 to 3.6]	3.2 [2.3 to 4.23]	89.96%
Iran (Islamic Republic of)	371.54 [262.57 to 449.49]	1766.41 [1509.85 to 2003.07]	3.11 [2.1 to 3.79]	3.16 [2.68 to 3.59]	375.42%
Kuwait	7.49 [5.95 to 9.34]	38.29 [29.2 to 53.21]	2.25 [1.78 to 2.81]	2.21 [1.67 to 3.04]	411.2%
Sudan	151.29 [105.05 to 198.35]	399.51 [298.6 to 515.96]	2.66 [1.79 to 3.46]	3.19 [2.35 to 4.14]	164.07%

In 2019, Qatar (178.86 [142.32 to 228.96]), Bahrain (165.78 [129.43 to 198.77]), and Oman (120.67 [95.12 to 145.85]) had the greatest ASDR owing to AFF. However, Kuwait (64.70 [49.47 to 84.46]), Libya (70.76 [51.83 to 91.33]), and Turkey (74.82 [57.84 to 95.30]) had the lowest ASDRs. The areas with the biggest increases in ASDR were Bahrain (%C = 65.4%), Morocco (%C = 17.1%), and Iraq (%C = 15.2%). Nonetheless, ASDR in Turkey (%C = -12.5%), Qatar (%C = -8.3%), and United Arab Emirates (%C = -4.3%) was substantially lower than in 1990 (Table 3).

**Table 3:** Age-Standardized Disability-Adjusted Rate (ASDR) for atrial fibrillation and flutter in Middle East and North Africa

Location Name	1990 Number (All ages)	2019 Number (All ages)	1990 ASDR [95%CI]	2019 ASDR [95%CI]	C% (Percentage Change)
Syrian Arab Republic	3543.83 [2749.52 to 4461.63]	8477.17 [6460.34 to 10815.65]	83.34 [65.11 to 103.79]	90.57 [69.49 to 113.06]	139.21%
Iran (Islamic Republic of)	13783.58 [10574.16 to 17796.09]	47997.33 [37710.42 to 60548.24]	75.31 [57.33 to 95.61]	75.3 [59.93 to 94.58]	248.22%
Global	3787838.26 [2961188.21 to 4832673.41]	8393635.01 [6693983.72 to 10541460.91]	110.0 [87.66 to 139.16]	107.13 [86.18 to 133.73]	121.59%
Iraq	5744.89 [4386.24 to 7901.16]	18169.49 [14107.4 to 24797.11]	83.87 [63.61 to 116.26]	96.62 [75.38 to 137.25]	216.27%
Tunisia	3058.67 [2403.65 to 3925.33]	9334.85 [7126.81 to 11851.38]	76.74 [61.13 to 97.05]	82.77 [63.74 to 104.79]	205.19%
Jordan	845.04 [666.48 to 1067.9]	4032.74 [3168.88 to 5062.98]	88.73 [70.68 to 110.64]	85.19 [68.51 to 104.14]	377.23%
Kuwait	306.39 [231.63 to 401.94]	1352.4 [1020.39 to 1791.8]	66.55 [50.97 to 85.8]	64.7 [49.47 to 84.46]	341.4%
Türkiye	25980.88 [20569.45 to 32275.05]	61728.56 [47405.24 to 79020.62]	85.52 [67.83 to 106.82]	74.82 [57.84 to 95.3]	137.59%
Palestine	631.23 [484.41 to 802.04]	1684.59 [1342.55 to 2146.63]	85.31 [65.53 to 108.51]	92.92 [74.6 to 117.47]	166.87%
Oman	432.46 [324.16 to 552.34]	1093.97 [871.07 to 1367.49]	112.66 [80.36 to 142.8]	120.67 [95.12 to 145.85]	152.97%
Lebanon	1596.79 [1264.11 to 2021.9]	4427.76 [3335.65 to 5559.39]	87.09 [69.39 to 109.06]	86.47 [65.25 to 108.85]	177.29%
Yemen	2670.18 [1965.36 to 3590.8]	7951.08 [5991.9 to 10322.47]	73.32 [54.02 to 96.44]	77.67 [59.98 to 99.56]	197.77%
United Arab Emirates	247.83 [179.27 to 339.85]	2213.92 [1469.86 to 3300.23]	94.05 [66.93 to 127.04]	90.02 [60.43 to 129.24]	793.33%
Libya	1066.3 [801.64 to 1413.51]	3094.51 [2247.74 to 3981.27]	66.48 [49.76 to 88.61]	70.76 [51.83 to 91.33]	190.21%
Morocco	8292.44 [6384.41 to 10647.75]	22499.69 [17709.74 to 28188.8]	76.25 [59.27 to 96.92]	89.32 [70.27 to 110.18]	171.33%
North Africa and Middle East	106968.06 [84330.27 to 135033.2]	289997.54 [229099.78 to 361786.29]	79.91 [63.99 to 99.66]	81.61 [65.1 to 100.78]	171.11%
Sudan	5411.92 [4112.74 to 7122.44]	11805.94 [8970.14 to 15214.9]	70.88 [54.38 to 92.57]	77.82 [59.82 to 99.82]	118.15%
Afghanistan	4051.69 [3018.64 to 5409.66]	7078.63 [5363.65 to 9385.66]	69.57 [51.66 to 91.18]	76.41 [59.48 to 98.79]	74.71%
Algeria	7955.75 [6164.21 to 10066.99]	24352.38 [18955.53 to 30289.51]	92.14 [73.11 to 115.68]	91.18 [71.78 to 111.62]	206.1%
Bahrain	121.42 [98.04 to 151.42]	945.88 [716.72 to 1174.38]	100.24 [82.24 to 126.38]	165.78 [129.43 to 198.77]	679.0%
Egypt	17440.09 [13077.6 to 22469.6]	40892.81 [29898.61 to 53997.19]	78.24 [59.11 to 98.86]	85.85 [62.32 to 110.67]	134.48%
Qatar	122.05 [92.55 to 148.91]	752.96 [565.01 to 974.58]	194.99 [142.12 to 238.95]	178.86 [142.32 to 228.96]	516.91%
Saudi Arabia	3592.68 [2628.11 to 4667.55]	9816.24 [7547.56 to 12762.88]	81.78 [59.92 to 105.68]	85.11 [66.98 to 105.14]	173.23%

### SDI and burden of atrial fibrillation and flutter

**Fig. 4:**
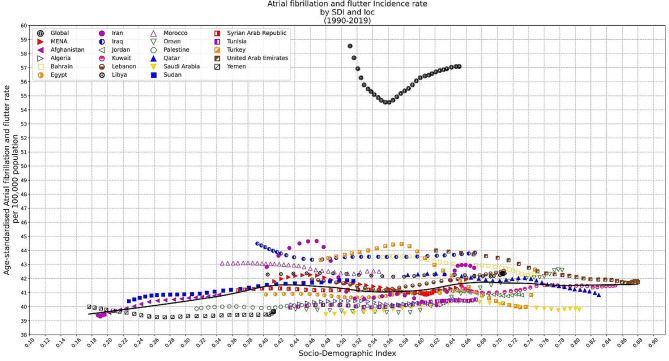
LOESS regression indicating the association between Age-standardized Incidence Rate of AFF and Socio-demographic Index (SDI) in MENA countries

**Fig. 5:**
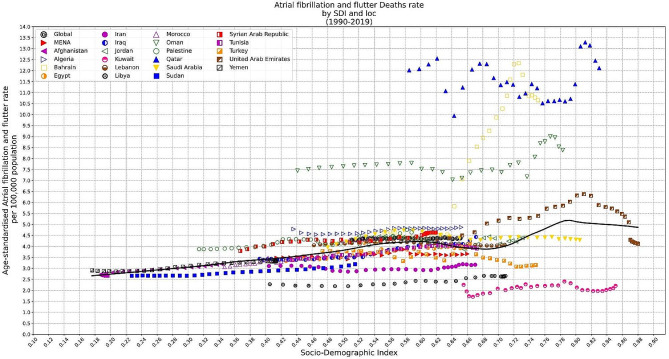
LOESS regression indicating the association between Age-standardized Mortality Rate of AFF and Socio-demographic Index (SDI) in MENA countries

In MENA, AFF’s ASIR seemed to have a neutral correlation to the SDI (Fig. 4). However, from SDI value of 0.18 to 0.42, the association was slightly positive. Regarding ASMR, LOESS regression line indicated a positive association till the SDI equal to 0.80, and then turned again into a neutral position (Fig. 5). This was somehow true about ASDR rates, too (Figure S3).

### Risk factors of mortality due to atrial fibrillation and flutter

The most important risk factor of mortality in AFF patients across the MENA region was high systolic blood pressure (SBP) (1.23 [0.93 to 1.59]) followed by high body mass index (BMI) (1.02 [0.60 to 1.61]), smoking (0.14 [0.09 to 0.2]), lead exposure (0.12 [0.07 to 0.17]), diet high in sodium (0.03 [0.01 to 0.12]), and alcohol use (0.02 [0.01 to 0.02]). Since 1990, ASMR due to high body mass index had the highest increase than other risk factors in MENA.

## Discussion

Through a secondary analysis of data from the Global Burden of Diseases Study 2019, the goal of this study is to comprehensively evaluate the regional burden of atrial fibrillation and atrial flutter in the Middle East and North Africa area, with a focus on ASMR, ASIR, and ASDR of AFF. The main findings of the study reveal that the ASIR of AFF in the MENA region showed minimal change over the past three decades, while a slight increase was observed in the ASMR and ASDR during the same period. Despite a consistent temporal trend, a small increase in ASIR and ASDR was noted between 2010 and 2019. Notably, AFF burden, as indicated by ASIR, ASMR, and ASDR, was consistently higher in females compared to males across the years, with age demonstrating a direct positive relationship with the burden of AFF. Additionally, Iraq, Iran, and Turkey were among the countries with the highest ASIR, while Qatar, Bahrain, and Oman had the highest ASMR and ASDR in 2019. Conversely, Kuwait, Libya, and Turkey exhibited the lowest ASMR and ASDR rates. The study also observed a nuanced correlation between the SDI and AFF burden, with high systolic blood pressure emerging as the most significant risk factor for mortality in AFF patients across the MENA region. Additionally, high body mass index showed the highest increase in ASMR since 1990 among the various risk factors assessed.

Comparing our findings to those found in global burden studies on AFF [15], the two studies found that the rates of incidence, mortality, and DALY associated with AFF have increased significantly, contributing to the global burden of the disease. Furthermore, age was found to be a key factor in determining the burden of AFF [15]. The trends for incidence, prevalence, deaths, and DALYs all increased with age, peaking at various age groups before progressively falling [15]. Moreover, atrial fibrosis, atrial hypertrophy, and conduction disorders are linked to aging and predispose older people to AFF [20]. Comorbid conditions such as heart failure, hypertension, and coronary artery disease, which are more common in elderly populations, further raise the risk of acquiring AFF as the population ages [21].

Our findings regarding gender disparities were in contrast to the global burden of AFF, which indicates a slightly higher overall burden for males [15]. This contrast may be explained with hormonal changes, especially those related to estrogen associated with menopause [22], that have been linked to the onset and development of AFF. It has been demonstrated that estrogen modulates cardiac electrical properties, such as atrial conduction and refractoriness, which may put women at risk for arrhythmias, including AFF [23]. Inequalities in healthcare and sociocultural variables could possibly be responsible for the gender differences in AFF burden in the MENA area. Gender norms and cultural practices have the potential to impact healthcare-seeking behaviors, treatment adherence, and access to preventative care [24]. Furthermore, the MENA region’s healthcare infrastructure might be better suited to handle cardiovascular diseases in men, which could result in underdiagnosis and undertreatment of AFF in women [25].

The intricate relationship between SDI and AFF burden in the context of the MENA area is influenced by a number of important elements. First, disparities in access to and infrastructure for healthcare are important. Higher SDI nations frequently have more developed healthcare systems, which include better-equipped hospitals, higher spending on healthcare, and more access to specialized medical services [26]. In light of this fact, people living in these nations might have better access to preventive care, prompt diagnosis, and effective treatment for cardiovascular diseases like AFF, which could lessen the burden overall. Socioeconomic differences within the MENA area can also affect how risk factors linked to AFF are distributed. Improved socioeconomic metrics, such as higher literacy rates, increased access to sanitary facilities and clean water, and improved nutrition, are frequently associated with higher SDI levels. In contrast, it is possible that lower SDI levels are associated with greater prevalence rates of risk factors such as diabetes, hypertension, and obesity, all of which are known to contribute to the onset and progression of AFF. Therefore, differences in the AFF burden among the MENA region’s nations may be partially explained by differences in SDI.

Identifying risk factors associated with mortality due to AFF is crucial for informing preventive strategies and clinical management. The leading risk factor is high SBP, which is indicative of the widespread impact of hypertension on cardiovascular health in the area. Increased risk of thromboembolic events, worsening myocardial ischemia, and accelerating the progression of cardiac remodeling are all established effects of elevated SBP that eventually have a negative impact on AFF patients [27]. With respect to the thromboembolic events, the age-standardized point prevalence and death rates of stroke in 2019 showed a decrease of 0.5% and 27.8% since 1990, respectively. Additionally, there was a 32.0% decrease in the regional age-standardized DALY rate in 2019 compared to 1990. In the year 2019, Afghanistan had the highest age-standardized DALY rates, while Lebanon had the lowest at 752.9. In terms of region, there were more stroke cases in the 60–64 age group and women had a higher prevalence of stroke in all age groups. Furthermore, there was an overall negative correlation between SDI and the burden of stroke from 1990 to 2019. Additionally, in the year 2019, the greatest burdens of stroke in the MENA region were attributed to high systolic blood pressure [53.5%], high body mass index [39.4%], and ambient particulate air pollution [27.1%] [28].

Furthermore, the high incidence of metabolic syndrome and obesity in the MENA population highlights the importance of having a high BMI as a mortality predictor [29]. Obesity rates among adults rose from 15.1% in 1980 to 20.7% in 2015. In the year 2015, high BMI in Eastern Mediterranean Region caused 417,115 deaths and 14,448,548 DALYs, representing approximately 10% and 6.3% of total deaths and DALYs across all age groups [30]. Obesity-related changes in structure and function play a role in causing AF through various pathways like excess fat, inflammation, fibrosis, oxidative stress, changes in ion channels, and dysfunction of the autonomic nervous system. Expanding epicardial adipose tissue during obesity is thought to be a main factor in the development of AF through paracrine signaling and direct infiltration [31]. Lack of physical activity is notable, particularly among women, along with an unhealthy diet that involves low intake of whole grains, nuts, and seafood [29].

The use of anticoagulants for the treatment of AFF in the MENA region has been evolving over the past few decades. Traditionally, vitamin K antagonists like warfarin were the mainstay of treatment [32]; however, their use is often complicated by the need for regular monitoring and dietary restrictions. In recent years, DOACs have gained popularity due to their predictable pharmacokinetic profiles and lack of need for routine monitoring [33]. Even though there are benefits, the use of DOACs in the MENA area varies due to factors like price, accessibility, and the knowledge of the physician. Furthermore, differences in healthcare facilities and patient knowledge play a role in the most effective utilization of anticoagulation treatment. Nevertheless, this treatment approach comes with a substantial chance of severe bleeding. Major bleeding risk factors in the MENA region are complex and include common comorbidities such as hypertension, diabetes, and renal impairment that are widespread among the population. Moreover, differences in healthcare infrastructure and availability of regular anticoagulation monitoring can increase the risk. Factors related to culture, diet, and the use of traditional remedies could affect the likelihood of bleeding.

Despite methodological improvements in the GBD 2019, the attainability of source data remained the biggest limitation in our study. Insufficient data entry and reporting can raise questions about the veracity of the information gathered. Lack of access to high-quality data may also skew study findings, which would affect the degree of evidence. Because there may be fewer healthcare facilities initiating the epidemiological studies, the effects may be more severe in nations with lower SDI and ongoing conflicts.

## Conclusion

The findings of this study shed light on the burden of atrial fibrillation and flutter (AFF) in the Middle East and North Africa (MENA) region. While the age-standardized incidence rate (ASIR) remained relatively stable over the past three decades, a slight increase was noted in both the age-standardized mortality rate (ASMR) and age-standardized disability-adjusted rate (ASDR) attributable to AFF. Females consistently exhibited higher ASIR, ASMR, and ASDR compared to males, with age demonstrating a direct positive relationship with the burden of AFF. National disparities were evident, with countries like Iraq, Iran, and Turkey reporting the highest ASIR, while others such as Yemen, Saudi Arabia, and Palestine had lower rates. Significant increases in ASIR were observed in Oman, Afghanistan, and Sudan, while reductions were noted in the United Arab Emirates, Qatar, and Bahrain since 1990. Bahrain, Morocco, and Iraq saw the largest increases in ASMR, whereas Turkey, United Arab Emirates, and Kuwait reported significant declines. High systolic blood pressure emerged as the most significant risk factor for mortality in AFF patients across the MENA region, underscoring the importance of targeted interventions to mitigate these risks and reduce the burden of AFF in the region.

### Electronic supplementary material

Below is the link to the electronic supplementary material.

Supplementary Material 1

## Data Availability

The datasets generated and analysed during the current study are available in the Global Burden of Diseases, Injuries, and Risk Factors Study (GBD) 2019, at https://vizhub.healthdata.org/gbd-results/.
