# Supplementary material for: The burden of atrial fibrillation/flutter in the Middle East and North Africa region and its associated risk factors from 1990 to 2019
Source: BMC Cardiovasc Disord. 2024 Jul 16;24:366. doi: 10.1186/s12872-024-04019-2 (PMC11251306; doi:10.1186/s12872-024-04019-2)
Supplement: Supplementary file 1 — Supplementary Material 1 [file 12872_2024_4019_MOESM1_ESM.docx]

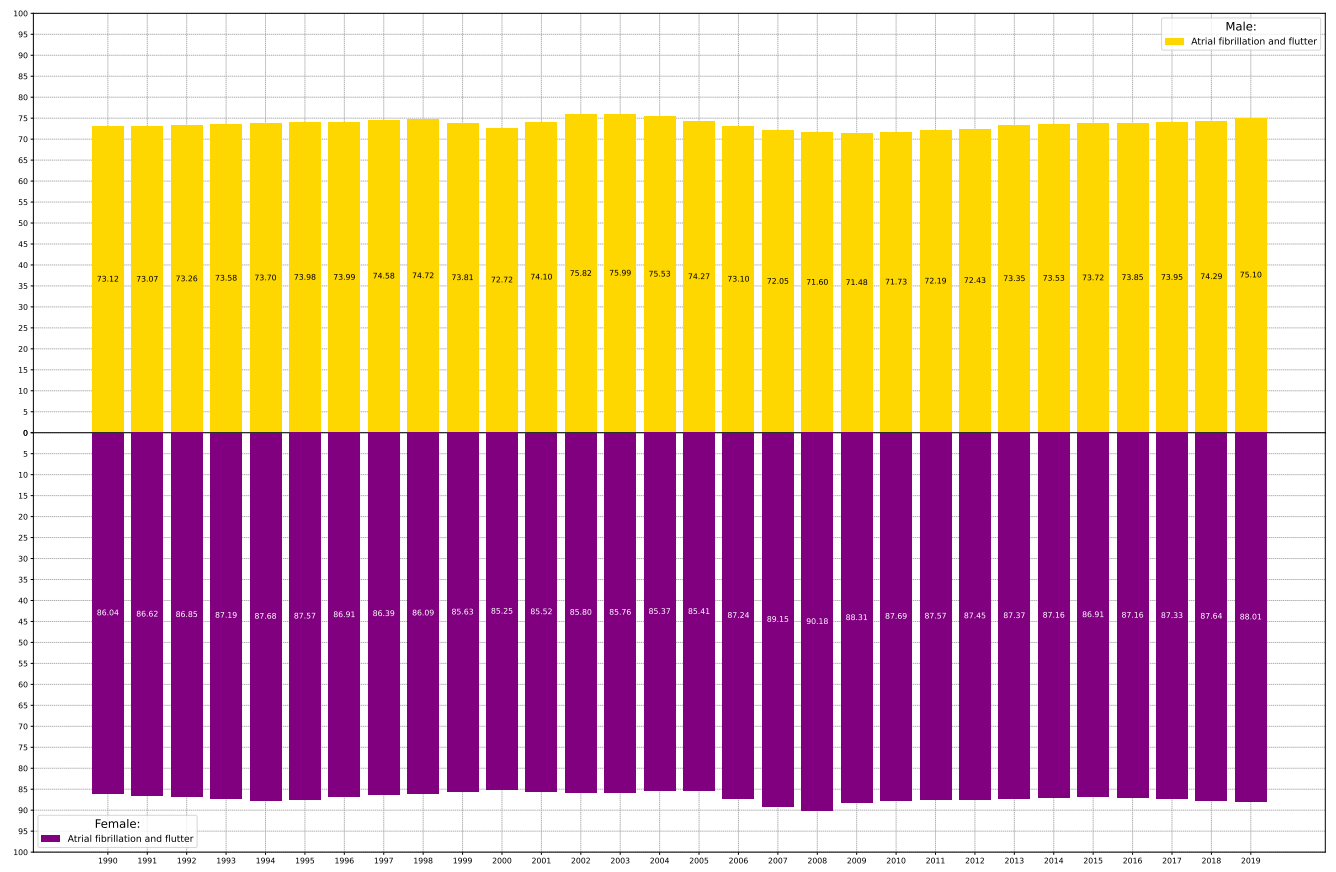


Figure S1. Age-Standardized Disability-Adjusted Rate of AFF from 1990 to 2019


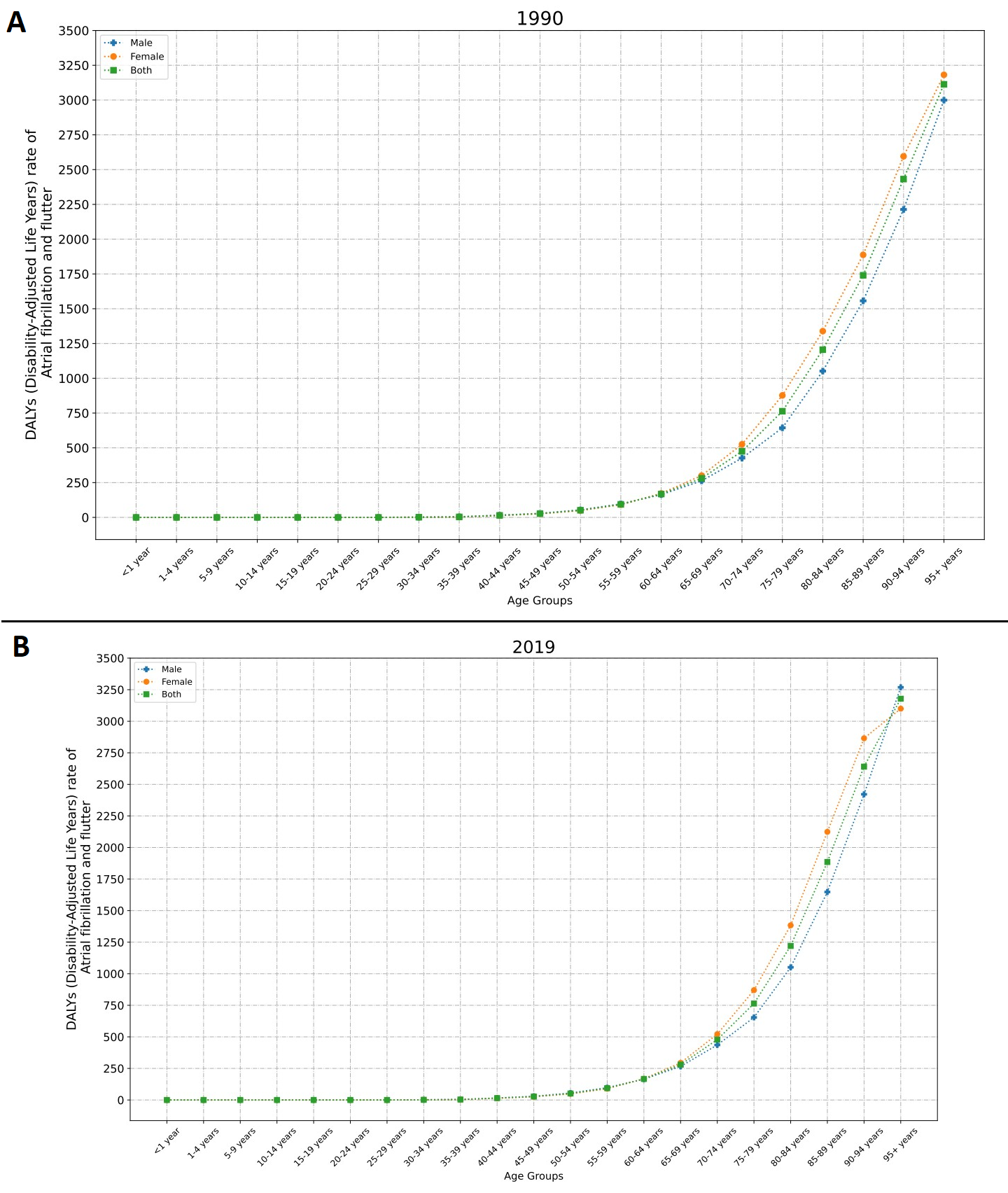


Figure S2. LOESS regression indicating the association between Age-Standardized Disability-Adjusted Rate of AFF


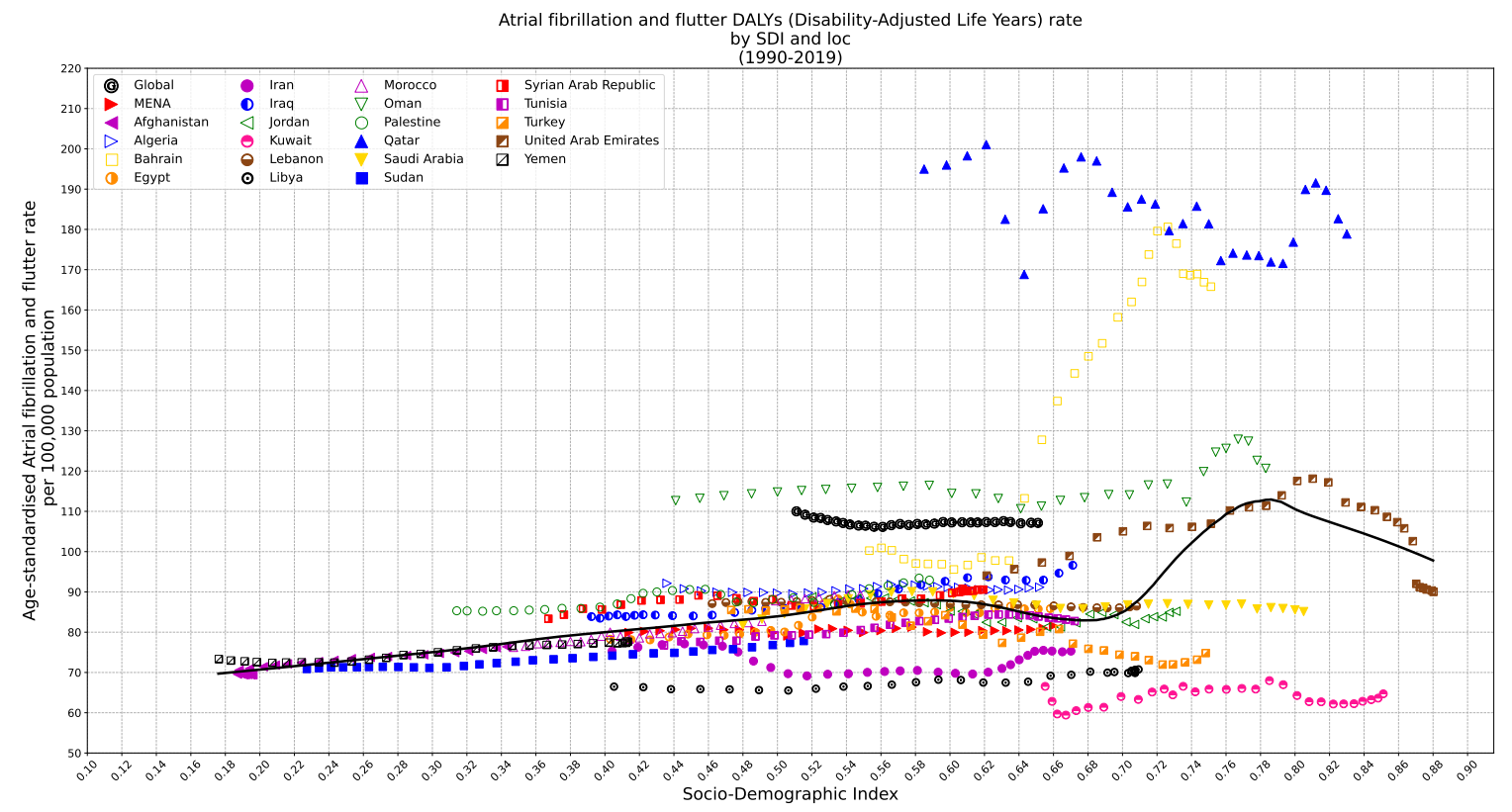


Figure S3. LOESS regression indicating the association between Age-Standardized Disability-Adjusted Rate of AFF and Socio-demographic Index (SDI) in MENA countries
